# Immune function analysis of *LsSd*, a transcription factor of the Hippo signaling pathway, in the cigarette beetle *Lasioderma serricorne*

**DOI:** 10.3389/fphys.2022.1042897

**Published:** 2022-10-11

**Authors:** Yue Zhang, Jiapeng Yang, Renhuai Dai, Wenjia Yang, Xiaomin Zhang

**Affiliations:** ^1^ Guizhou Provincial Key Laboratory for Agricultural Pest Management of the Mountainous Region, Institute of Entomology, Guizhou University, Guiyang, China; ^2^ Guizhou Provincial Key Laboratory for Rare Animal and Economic Insect of the Mountainous Region, College of Biology and Environmental Engineering, Guiyang University, Guiyang, China; ^3^ China Tobacco Guizhou Industrial Co. Ltd, Guiyang, China

**Keywords:** *Lasioderma serricorne*, *LsSd* gene, Hippo-signaling pathway, wing development, immune response

## Abstract

The Scalloped (Sd) is a transcription factor that regulates organ size control in the Hippo-signaling pathway. Recent studies have showed that Hippo signaling also functions in the innate immune response. Although the Sd gene has been reported in many insects, their immune functions remain unexplored. In this study, the *LsSd* gene of *Lasioderma serricorne*, with a complete open reading frame that encodes a protein composed of 402 amino acids was identified. *LsSd* was predominantly expressed in early pupae. Tissue-specific analyses revealed that the highest concentrations of *LsSd* were detected in the midgut and brain. At 1–24 h after *Escherichia coli* infection, *LsSd* expression increased substantially. However, *LsSd* expression was downregulated 3–12 h after *Staphylococcus aureus* infection. RNA interference-mediated silencing of the *LsSd* transcript resulted in deformed, considerably smaller, and degenerated wings. Meanwhile, *LsCycE*, *LsDiap1*, and *LsVg*, which are involved in cell proliferation and wing development, were drastically reduced when *LsSd* was depleted. In a survival assay, the *LsSd* knockdown considerably decreased the susceptibility to *S. aureus*, a gram-positive bacterium. In addition, knockdown of *LsSd* remarkably downregulated the transcription of *LsCact* in response to *S. aureus* stimulation, while upregulating the expression of five immune-related genes. Our results provide conclusive proof for the important roles of *LsSd* in the immune response of *L. serricorne*.

## Introduction

The Hippo signaling pathway is a conserved signal transduction network that regulates organ size in a wide range of animal species (Misra and Irvine, 2018). In *Drosophila melanogaster*, the core of this pathway is composed of four proteins: the kinases Hippo and Warts (Wts) and their cofactors Salvador and Mob as a tumor suppressor (Liu et al., 2016). Genetic screening revealed that they were initially identified as *Drosophila* tumor suppressors. Loss-of-function mutations in any of these four genes dysregulate tissue growth and development in *D. melanogaster* (Staley and Irvine, 2012). Yorkie (Yki) is the central effector of the Hippo signaling pathway and plays a crucial role in the regulation of cell proliferation and apoptosis. Activation of this signaling cascade will phosphorylate the transcription co-activator Yki in order to promote apoptosis and limit organ size overgrowth (Zhao et al., 2011). When the Hippo signaling pathway kinase cascade is inactivated, Yki accumulates in the nucleus and binds to transcription factors to promote the expression of target genes involved in cell growth, proliferation, and survival.

The wing plays a crucial role in the environmental adaptation of insects. It enables insects to move efficiently for dispersal, feeding, mating, and escape from unfavorable environments and predators (Ye et al., 2022). Studies have shown that the Hippo-signaling pathway is necessary for wing formation during *D. melanogaster* development. For instance, mutations of *Wts* that increase Yki activity result in wing overgrowth, whereas loss of Yki activity decreases wing growth (Pan et al., 2018). Scalloped (Sd), a transcription factor, is a critical partner of Yki. It forms a transcriptional complex with Yki to promote cell growth and proliferation (Zhang et al., 2008). Sd can mediate various functions by interacting with tissue-specific co-factors, such as Vestigial (Vg), *D. melanogaster* homologue of Myocyte enhancer factor-2 (Dmef2), and Nerfin-1 (Bandura and Edgar, 2008; Deng et al., 2009; Vissers et al., 2018). Loss of Sd function results in Yki-dependent transcription and organ size reduction, whereas Sd activation promotes tissue overgrowths. *Sd* mutations in *D. melanogaster* result in abnormal wings with ectopic bristles and wing margin gaps (Campbell et al., 1991; Simmonds et al., 1998). In *Bombyx mori*, *BmSd* knockdown or knockout resulted in small, curled wings (Yin et al., 2020). Similarly, in *Locusta migratoria*, gene silencing of *LmSd* by RNA interference (RNAi) resulted in deformed wings (Zhang et al., 2021a). In addition to its function in wing development, Sd affects the development of other *D. melanogaster* organs, including the eyes, legs, optic lobe, and lymph glands (Garg et al., 2007; Ferguson and Martinez-Agosto, 2017).

Surprisingly, the Hippo signaling pathway is also associated with immune responses. In *D. melanogaster*, the Hippo–Yki pathway is required for the regulation of innate immunity. Loss of suppressors of the Hippo signaling pathway tumor or activation of Yki in fat bodies causes elevated *Cactus* (*Cact*) mRNA levels, which inhibits Toll receptor-mediated antimicrobial response and increases susceptibility to gram-positive bacterial infection. Furthermore, gram-positive bacteria activate Hippo–Yki signaling *via* the Toll-Myd88-Pelle cascade through Pelle-mediated phosphorylation and degradation of the Cka subunit of the STRIPAK PP2A complex (Liu et al., 2016). In the Chinese crab (*Eriocheir sinensis*), both gram-positive and gram-negative bacteria induced the activation of Hippo signaling and inhibited the expression of *Cact*, causing the nuclear accumulation of Dorsal from the Toll pathway to upregulate the expression of antimicrobial peptide genes (Yang et al., 2019). In conclusion, the aforementioned studies demonstrated that the Hippo signaling pathway is essential for the regulation of antibacterial immunity.

The cigarette beetle, *L. serricorne* (Fabricius), is a widespread and destructive storage pest worldwide due to its high reproductive potential and adaptability (Kim et al., 2003; Mahroof and Phillips, 2008). It causes damage to tobacco products, cereal grains, materials used in Chinese medicine, and processed foods (Abdelghany et al., 2016). It can cause massive economic damage to stored materials through larval feeding and feces production (Riudavets et al., 2007). In general, the effective management of *L. serricorne* relies heavily on the use of phosphine-containing fumigants, which has led to several risks, including environmental pollution, pest resurgence, and the development of insecticide resistance (Saglam et al., 2015). Owing to these detrimental effects, innovative and long-term control strategies are required. RNAi has considerable potential to prevent pest damage to stored food (Zhu and Palli, 2020). Given the importance of *Sd* in the Hippo-signaling pathway, it is anticipated that *Sd* will become an alternative molecular target of RNAi, thereby reducing the use of pesticides in pest control.

In this study, we identified the orthologs of *Sd* from *L. serricorne* (named as *LsSd*) and determined its mRNA profiles and gene functions in wing development and innate immune. These results will clarify the roles of *LsSd* in the immune response of *L. serricorne* and lay a foundation for further research on the functions of *LsSd*.

## Materials and methods

### Insect rearing

*L. serricorne* adults were collected from a tobacco warehouse in Guiyang city, Guizhou province, China, in the fall of 2016. The larvae were fed a mixture of crushed maize, beer yeast*,* and dried tobacco. The beetles were kept in an insectary at 25 ± 1°C under a 16 h: 8 h light-dark photoperiod and 60 ± 5% relative humidity (RH).

### Gene cloning and sequence analysis

*LsSd* gene was identified by local-tBlastn searches using homolog sequences from *Tenebrio molitor*, *Diabrotica virgifera,* or *Tribolium castaneum* as queries against the *L. serricorne* pupae transcriptome (SRR13065789). *LsSd* cDNA were amplified by reverse transcriptase polymerase chain reaction (RT-PCR) using the *LsSd*-specific primers (Supplementary Table S1). Thermal cycling was carried out in a thermocycler (T100 Thermal Cycler, Bio-Rad, Hercules, CA, United States) with the following reaction program: 94°C for 3 min; 34 cycles of 94°C for 30 s, 57°C for 30 s, and elongation at 72°C for 1.5 min; and then an extension at 72°C for 10 min. Amplified PCR product was purified using a DiaSpim Column DNA Gel Extraction Kit (B110092, Sangon Bioech, China) and sequenced in both directions by Sangon Bioech.

The NCBI ORF finder (https://www.ncbi.nlm.nih.gov/orffinder/) was used to predict the open reading frame and protein sequence of *LsSd*. DNAMAN 6.0 software (LynnonBiosoft, Vaudreuil, Quebec, Canada) was used to analyze the cDNA sequence and deduce the amino acid sequence. Domain structures were determined by using ProCite (https://prosite.expasy.org) and SMART (http://smart.embl-heidelberg.de/smart/) servers. The molecular weight and isoelectric point were computed by the ExPASy Proteomics Server (http://www.expasy.ch). Using Clustal W, the amino acid sequences deduced for the Sds were aligned. Phylogenetic analysis and phylogenetic tree construction were performed using MEGA Version 6 with the neighbor-joining (NJ) method and a p-distance model with 1,000 bootstrap replicates.

### Expression pattern of *LsSd*

RT-qPCR was used to determine the expression of *LsSd* in different developmental stages (early larvae (mixture of 1-2 old days), late larvae (30-day-old), early pupae (1-day-old), late pupae (5-day-old), early adults (1-day-old), and late adults (7-day-old)), different tissues from the late larvae (brain, integument, fat body, midguts, and Malpighian tubules) and different tissues from the early adults (head, thorax, abdomen, and wings) were used.

Gram-positive bacterium *S. aureus* (ATCC25923) and Gram-negative bacterium *E. coli* (ATCC25922) were purchased from Solarbio (Beijing, China). The bacterial cells were cultured in Luria-Bertani (LB) broth at 37°C. *S. aureus* and *E. coli* were cultured until their respective optical densities reached approximately 0.6 and 0.5 at 600 nm. Each late-stage larva was injected with 100 nL of the pathogen suspensions or endotoxin-free sterile water according to the method described previously (Yan et al., 2021). The larvae were transferred to an artificial diet and maintained in 9 cm diameter Petri dishes) The samples were collected at 1, 3, 6, 12, and 24 h after injection. Each treatment consisted of three replicates, and each replicate comprised a pool of 10 individuals. The total RNA was extracted from *L. serricorne* larvae using HP Total RNA kit (Omega Bio-Tek, Norcross, GA, United States). One microgram of total RNA was reverse-transcribed in a 20 μL reaction mixture containing the HiFiScript gDNA Removal cDNA Synthesis Kit (CW2582M, CWBIO, China) according to the manufacturer’s protocols. The RT-qPCR analysis was conducted with FastStart Essential DNA Green Master Mix (Roche, Indianapolis, IN, United States) and the primers listed in Supplementary Table S1. The thermal cycling was carried out in a CFX96 Touch™ Real-Time PCR Detection System (Bio-Rad, Hercules, CA, United States) under the following reaction conditions: 95°C for 10 min, then 40 cycles (95°C for 30 s and 60°C for 30 s), followed by melting from 65°C to 95°C. The *RPL13a* gene served as reference gene (Zhang et al., 2021b). The relative expression levels of the *LsSd* gene were determined using the 2^−ΔΔCt^ method (Livak and Schmittgen, 2001). There were three biological replicates and three technical replicates for each biological replicate.

### Functional analysis based on RNA interference

The *LsSd* targets fragment for RNAi was amplified by PCR using the gene-specific primers ds-*LsSd*-F and ds-*LsSd*-R (Supplementary Table S1). The double-stranded RNA (dsRNA) targeting the *LsSd* gene were prepared *in vitro* using the TranscriptAid T7 High Yield Transcription Kit (Thermo Scientific, Waltham, MA, United States). The purified dsRNA was quantified using a NanoDrop 2000 spectrophotometer (Thermo Scientific, Waltham, MA, United States). The quality and molecular size of dsRNA products were determined in a 1.2% agarose gel. *GFP* dsRNA (a nonspecific negative control) was synthesized as described previously (Yang et al., 2020).

To investigate the roles of *LsSd* in *L. serricorne* development, 300 ng gene-specific dsRNA was injected into the body cavity of each late larvae or early pupae. Ten larvae were sampled between 12 and 72 h after dsRNA injection. The efficiency of RNAi was detected by RT-qPCR, as described above. Post-injection insects were reared under the same conditions, as mentioned above, to observe their growth, development, and phenotypes. After *LsSd* knock down by dsRNA injection for 24 h, the relative expression levels of one wing development-related gene (*LsVg*) and two genes related to cell proliferation and death (*LsCycE* and *LsDiap1*) were analyzed by RT-qPCR as described above.

To further investigate the role of *LsSd* in immune defense, we injected ds-*LsSd* into late larvae and stimulated them with 200 nL of bacterial suspension (*S. aureus* OD_600_ = 1.2 or *E. coli* OD_600_ = 1) 24 h later using the previously described method. Transcript levels of *LsSd*, *LsCact* and six immune-related genes (*LsAtt2*, *LsDef1*, *LsDef2*, *LsCole*, *LsLysC*, and *LsLysI*) were determined at 12 h using RT-qPCR. Each group of fifty insects was to be observed for 5 days at 1-day intervals. Each experiment involved three biological replications.

### Statistical analysis

The statistical analyses and graphs were generated using GraphPad Prism 8 software (GraphPad Software, San Diego, CA, United States). One-way analysis of variance (ANOVA) was followed by a Tukey HSD test was used to compare the gene expression level of *LsSd* under each normalization treatments. The Student’s *t*-test was used to determine statistical significance between treatment group and the control group. The Kaplan-Meir log-rank test was used to examine survival rates. *p* < 0.01 was regarded as extremely significant, while value of *p* < 0.05 was considered statistically significant.

## Results

### Bioinformatics analysis of the *LsSd* sequence

**FIGURE 1:**
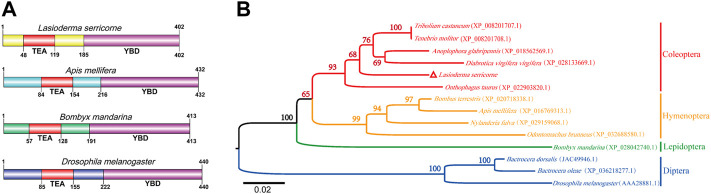
Phylogenetic tree and predicted structure of the Sd proteins in insects. **(A)** The deduced amino acid sequences were used to predict the domain of Sd proteins using SMART tool. **(B)** The phylogenetic tree of Sd protein was constructed by MEGA6 software using the neighbor-joining method with 1,000 bootstrap replications. Numbers at the branch nodes represent the level of bootstrap support for each branch.

**FIGURE 2:**
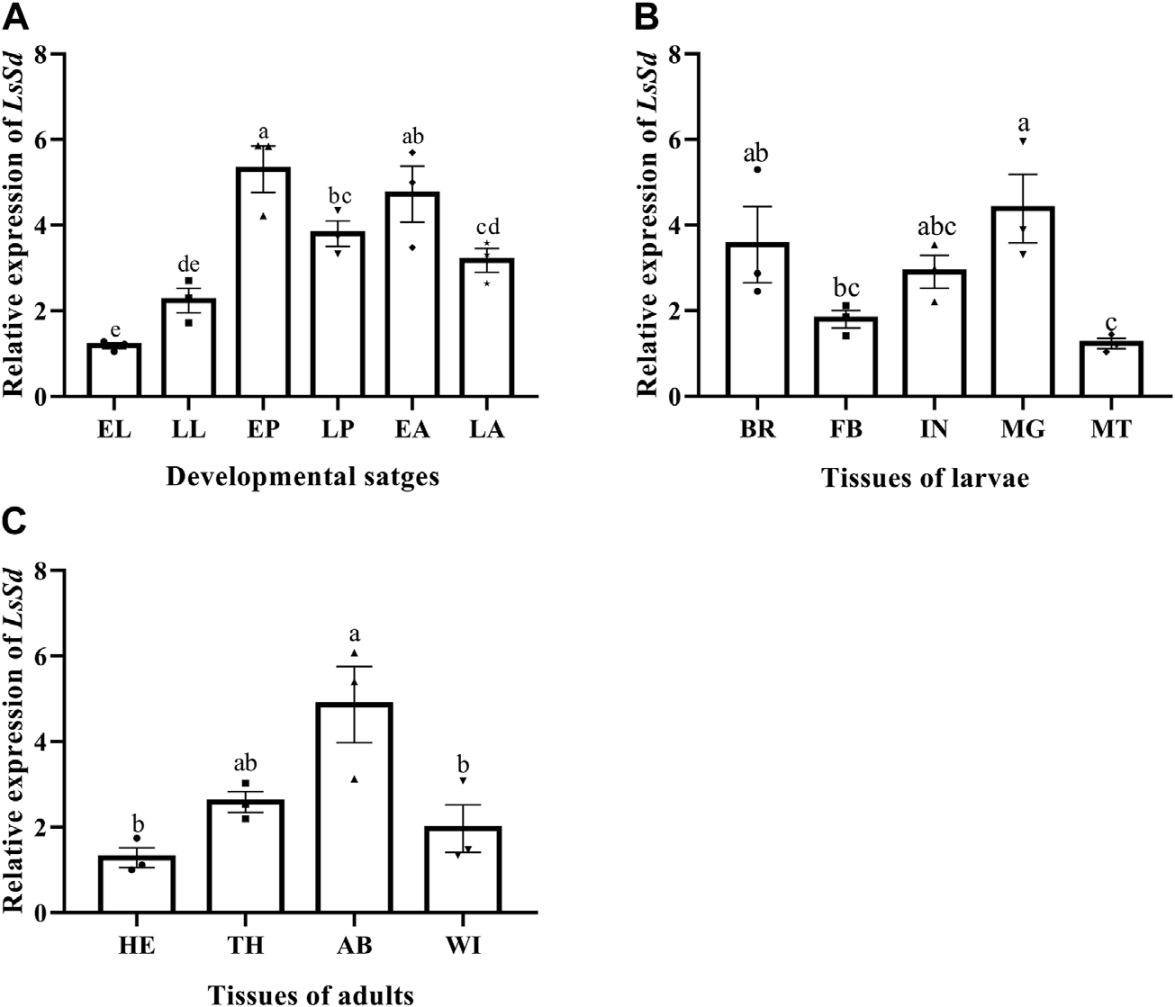
*LsSd* expression in the different developmental stages **(A)**, different larval tissues **(B)** and different adult tissues **(C)** of *L. serricorne*. EL, early larvae; LL, late larvae; EP, early pupae; LP, late pupae; EA, early adults; LA, late adults; BA, brain; FB, fat body; IN, integument; MG, midgut; MT, Malpighian tubules; HE, head; TH, thorax; AB, abdomen; Wing, WI. Error bars indicate the SEM of three independent biological replications. Different letters above the bars show significant differences at the 0.05 level (one-way ANOVA followed by Tukey’s test).

The putative cDNA sequence of LsSd was identified and validated using reverse transcriptase polymerase chain reaction (RT-PCR) from the *L. serricorne* pupae transcriptome. The open reading frame (ORF) contained 1,209 base pairs (bp) and encoded a polypeptide with 402 amino acids, a calculated molecular mass of 45.301 kDa, and an isoelectric point (pI) of 6.81. The ORF sequence (GenBank accession number: ON533736) was submitted to GenBank. Similar to *Apis mellifera*, *Bombyx mandarina*, and *D. melanogaster*, the amino acid sequence of LsSd contains an N-terminal TEA/ATTS (TEA) domain and a C-terminal YAP binding domain (YBD) (Figure 2A). Multiple sequence alignments showed that insect Sd proteins are highly conserved. (Supplementary Figure S1). To illustrate the molecular evolution of Sd, 14 mature Sd protein sequences were aligned to generate a neighbor-joining (NJ) tree. The dendrogram showed that Sd proteins of insects in each order clustered on a single branch (Figure 1B).

### Expression pattern analysis of *LsSd*

*LsSd* mRNA among developmental stages, different larvae tissues, and different adult tissues of *L. serricorne* was determined by RT-qPCR. mRNA levels of *LsSd* were widely expressed in diverse tissues and developmental stages (Figure 2). The *LsSd* transcription levels were considerably higher in pupae and adults than in larval instars (Figure 2A). The expression levels of *LsSd* were the highest in the midgut and brain compared with all other analyzed larval tissues. Only slight expression was detected in Malpighian tubules (Figure 2B). The expression levels of *LsSd* mRNA were found to be the highest in the abdomen of adult *L. serricorne*, followed by the thorax, wings, and head (Figure 2C).

**FIGURE 3:**
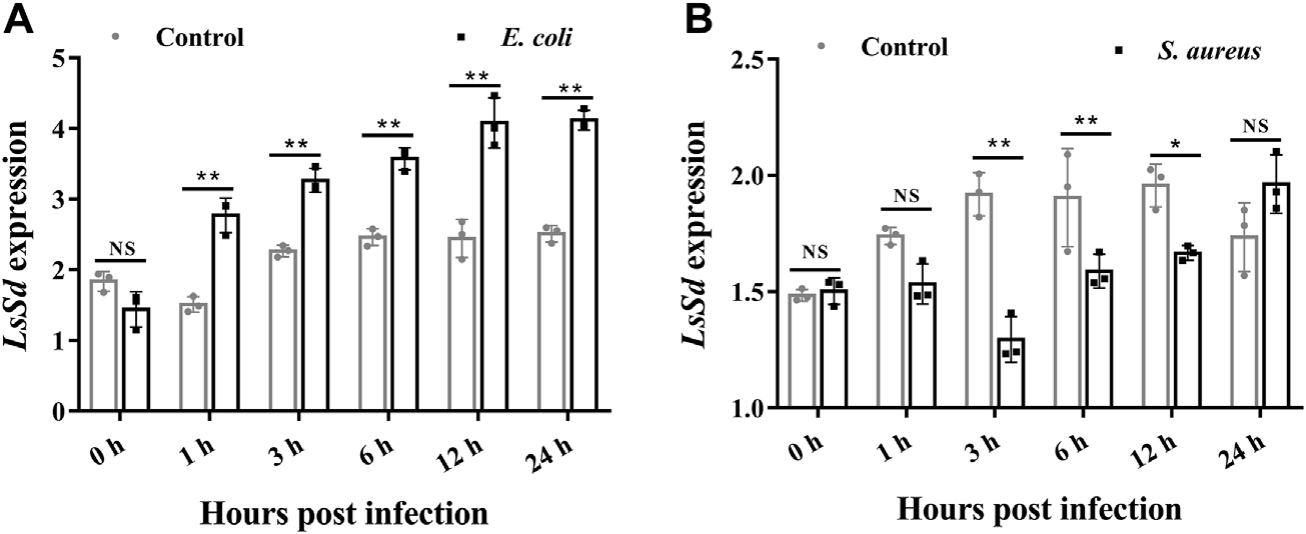
The mRNA transcript levels of *LsSd* in whole larvae (30-day-old) after microbial challenge. After injections of *E. coli* **(A)** and *S. aureus* **(B)**, the mRNA transcript levels of *LsSd* were analyzed by RT-qPCR. Error bars indicate the SEM of three independent biological replications. Significant differences between the microbial challenge group and control group were determined using Student’s *t*-test (*p < 0.05, **p < 0.01).

Changes in *LsSd* mRNA expression levels in response to immune challenges with *E. coli* and *S. aureus* were measured (Figure 3). The *LsSd* mRNA expression levels are upregulated 1–24 h after an *E. coli* challenge, but decrease 3–12 h after an *S. aureus* infection, according to our findings. This finding suggests a putative role of *LsSd* in systemic immune signaling.

### Functional analysis of *LsSd* based on RNAi

**FIGURE 4:**
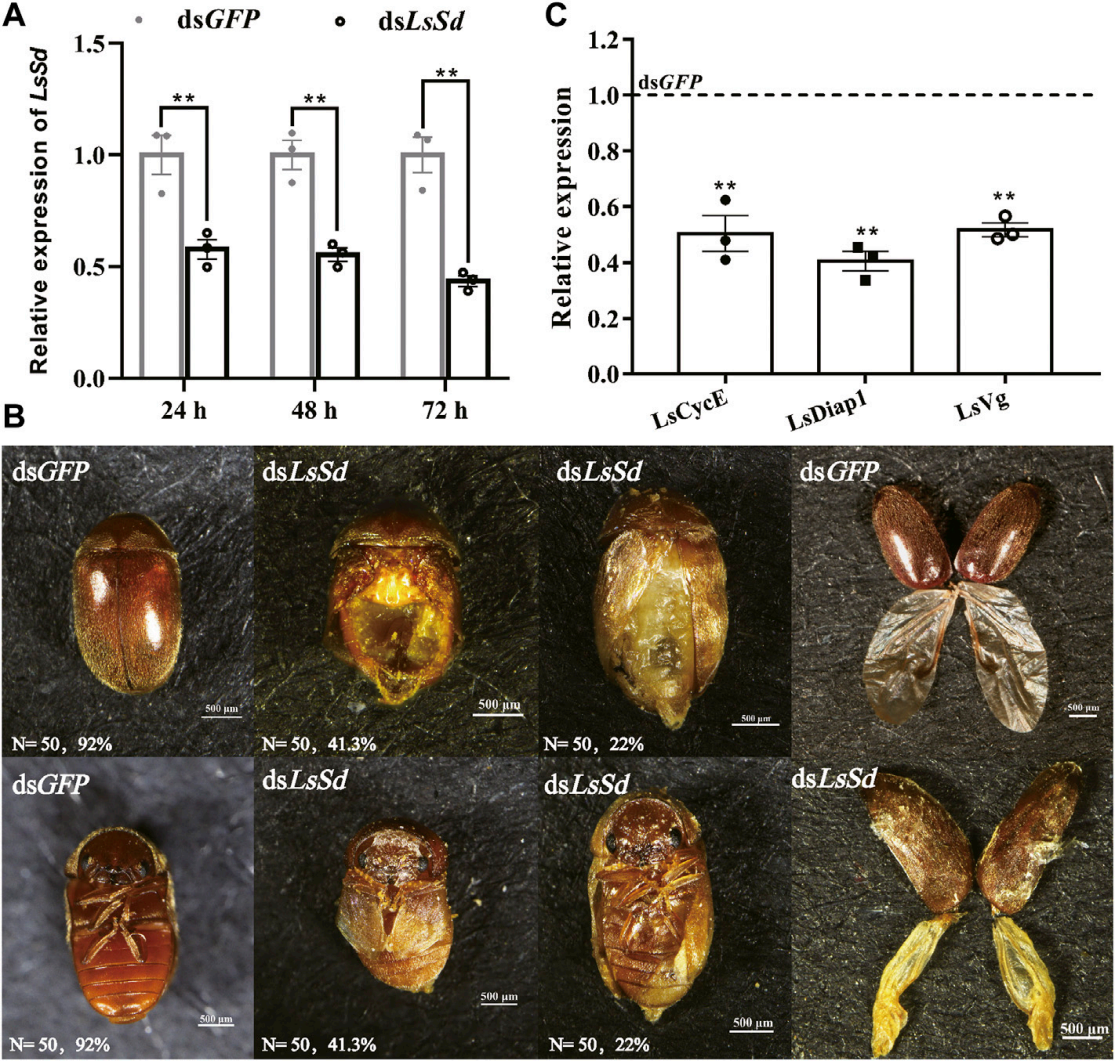
The effect of *LsSd* knockdown on adult phenotypic characterization in *L. serricorne*. **(A)** The expression levels of the *LsSd* gene after RNAi treatment in *L. serricorne* pupae. **(B)** Dorsal and ventral views of adults treated with ds*LsSd* and ds*GFP*, and structural comparison of wings after *LsSd* or *GFP* dsRNA injection. **(C)** The expression levels of genes related to cell proliferation and wing were examined by RT-qPCR after injection of ds*LsSd* in 24 h. Error bars indicate the SEM of three independent biological replications. Significant differences between control group and RNAi group were determined by Student’s *t* test. **p < 0.01.

To investigate the biological function of *LsSd* in *L. serricorne* wing development, dsRNA for *LsSd* and ds*GFP* were injected into early pupae. The RT-qPCR results showed that the transcription level was considerably reduced in comparison with dsGFP-injected pupae. After 72 h of treatment with ds*LsSd*, the RNAi efficiency was 56% compared with injections of *GFP* dsRNA (Figure 4A). RNAi in embryos can cause morphological defects in adults. Adult elytra and hindwings exhibited deformation in ds*LsSd*-injected individuals. Compared with the ds*GFP*-injected adults, the deformed elytra were smaller and more fragile. Meanwhile, the hindwings were curved and wrinkled (Figure 4B). To further investigate the causes of the changes in the wing structure and morphology of *L. serricorne* following *LsSd* RNAi, the expression levels of cell division and death genes were analyzed by RT-qPCR. The expression levels of *LsCyclinE* (*LsCycE*), *LsDiap1*, and *LsVestigial* (*LsVg*) were considerably lower than those of the control group (Figure 4C).

### Knockdown of *LsSd* promoted the survival rate of *L. serricorne*

**FIGURE 5:**
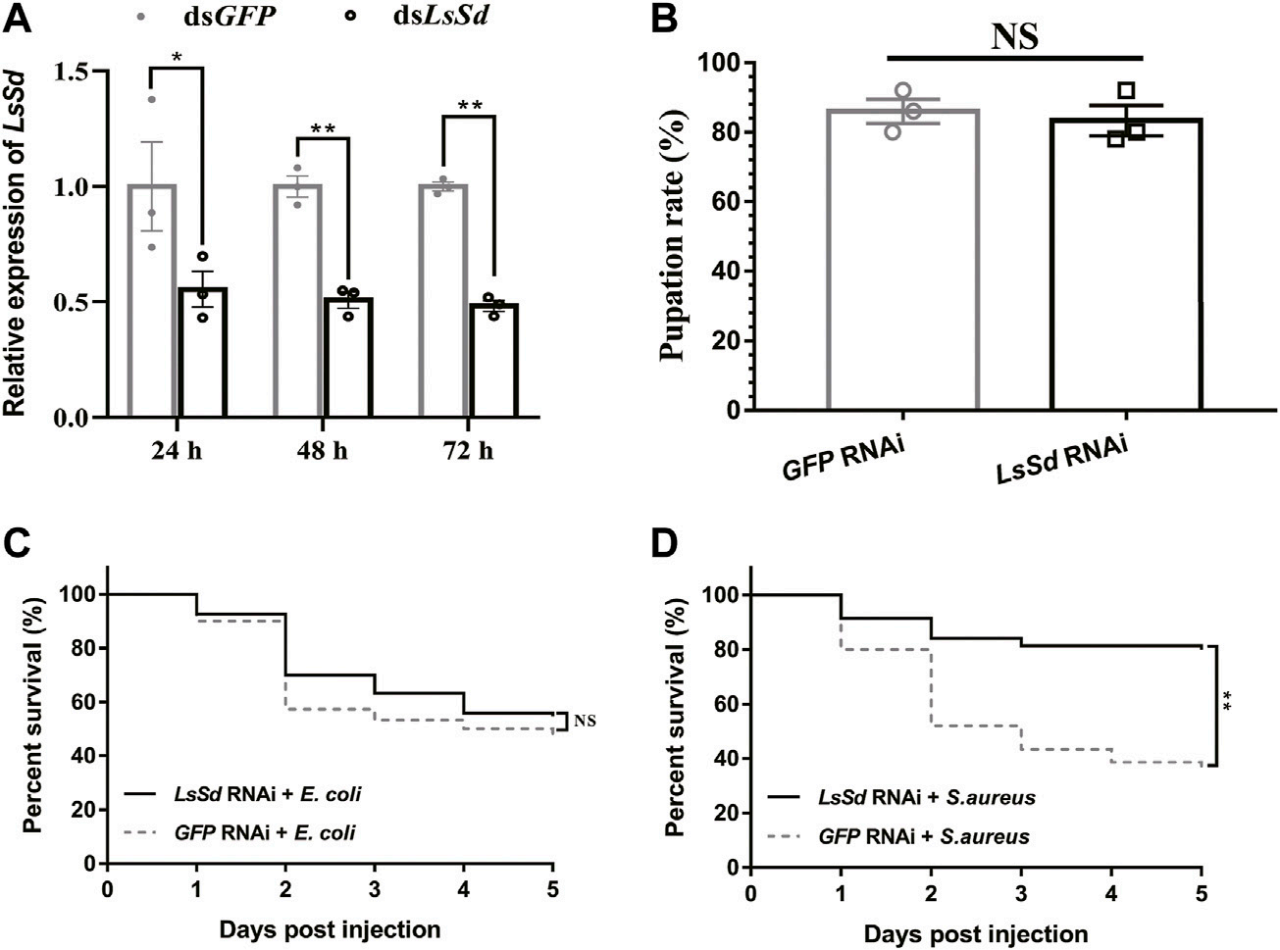
Susceptibility of *L. serricorne* larvae against *E. coli* and *S. aureus* infections after knockdown of *LsSd*. **(A)** RNAi efficiency of the *LsSd* gene was detected at 24, 48, and 72 h after dsRNA treatment in *L. serricorne* larvae. **(B)** Effect of *LsSd* RNAi on *L. serricorne* pupation rate. **(C)** *E. coli* and **(D)** *S. aureus* were inoculated at 24 h post *LsSd* RNAi, and larval survival was recorded daily for 5 days in each treatment group. Error bars indicate the SEM of three independent biological replications. Statistical analysis of survival analysis was carried out based on Kaplan-Meier method (Log-rank Chi-square test; **p < 0.01).

To determine the role of *LsSd* in innate immunity, dsRNA was injected into *L. serricorne* larvae, which effectively inhibited *LsSd* expression (Figure 5A). There were no statistically significant differences between the pupation rates of ds*LsSd*-injected and control larvae (Figure 5B). Next, we challenged *LsSd* knockdown larvae with *E. coli* or *S. aureus* and then assessed the survival of these larvae for 5 days. There was no significant difference in survival between *LsSd* knockdown larvae infected with *E. coli* and controls (Figure 5C). However, *LsSd* RNAi-treated larvae exhibited decreased sensitivity to the gram-positive bacterium *S. aureus* (Figure 5D).

### *LsSd* regulated AMPs’ expression *via LsCact*

**FIGURE 6:**
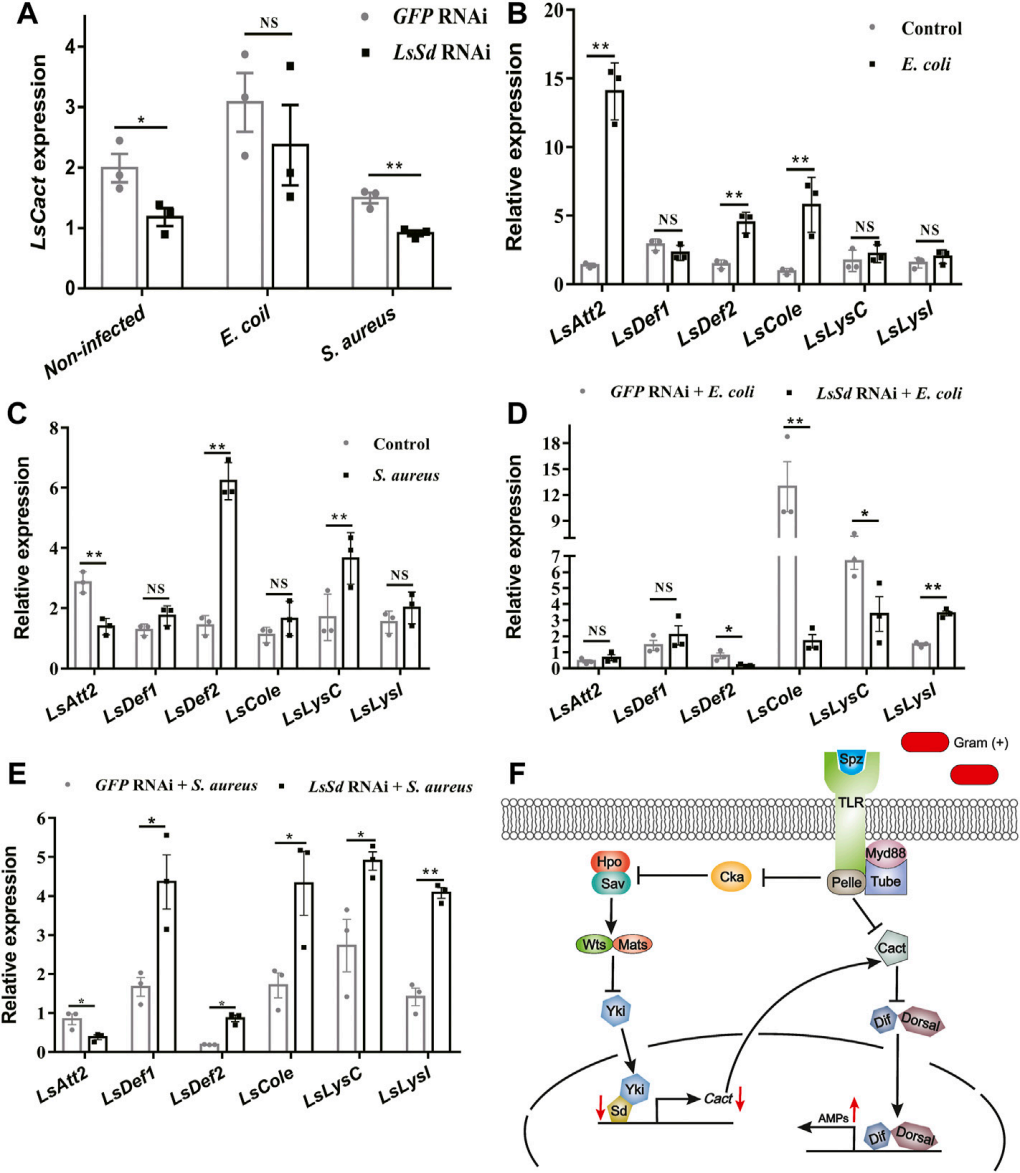
*LsSd* regulates production of anti-microbial peptides by acting *LsCact*. **(A)** *LsSd*-silenced larvae were treated with or without *E. coli* and *S. aureus* stimulations. The mRNA transcript levels of *LsCact* were analyzed at 12 h post-stimulation using RT-qPCR. After challenge injections of **(B)** *E. coli* **(C)** and *S. aureus* for 12 h, the mRNA transcript levels of six antimicrobial peptide genes were analyzed by RT-qPCR. **(D)** *E. coli* and **(E)** *S. aureus* were used to stimulate *LsSd*-silenced larvae. RT-qPCR was used to determine AMPs’ expression at 12 h post-stimulation. **(F)** Schematic representation based on previous studies (Liu et al., 2016; Huang et al., 2022) for *LsSd*-mediated regulation of AMPs in response to the Gram-positive bacteria *S. aureus*. Error bars indicate the SEM of three independent biological replications. Asterisks above bars indicate significant differences (*p < 0.05; **p < 0.01) analyzed by a Student’s *t* test.

It is demonstrated that *Cact* is a direct target of the Yki-Sd transcription factor complex (Liu et al., 2016). To determine whether *LsSd* also influenced AMP expression *via LsCact* in *L. serricorne*, we measured the transcript levels of *LsCact* in *LsSd*-silenced whole larvae following microbial challenge. After *S. aureus* infection of larvae injected with ds*LsSd*, the expression level of *LsCact* was considerably reduced (Figure 6A). In contrast, *LsSd* knockdown has no influence on *LsCact* expression under *E. coli*-challenged conditions (Figure 6A). Then, we determined the expression levels of six AMPs in *L. serricorne* larvae in response to a bacterial challenge (Figure 6B). The expression of *LsAtt2*, *LsDef2*, and *LsCole* was strong induced after *E. coli* infection (Figure 6B). The expression levels of *LsDef2* and *LsLysC* were considerably upregulated following *S. aureus* infection (Figure 6C). These results demonstrated that such AMPs are necessary for host defense against infection. To determine whether *LsSd* regulated the expression of AMPs, we knocked down *LsSd* expression in larvae. This resulted in a significant upregulation of the expressions of *LsDef1*, *LsDef2*, *LsCole*, *LsLysC*, and *LsLysI* in response to *S. aureus* stimulation (Figure 6E). In contrast, *LsSd* knockdown decreased *LsDef2*, *LsCole*, and *LsLysC* expression in *E. coli*-challenged conditions (Figure 6D). These findings support the hypothesis that *LsSd* knockdown reduces susceptibility to *S. aureus*. Our findings imply that *LsSd* knockdown affected the transcription of *LsCact* and five immune-related genes (Figure 6F).

## Discussion

Insect wings are an important organ for successful radiation of this animal group. Insect wing development is a complex process that requires coordinated regulation of multiple signaling pathways, including Hippo, c-Jun N-terminal kinase (JNK), hedgehog (Hh), and Notch (Glise et al., 2002; Lin et al., 2016; Pan et al., 2018). Sd, a key component of the Hippo-signaling pathway, plays a crucial role in wing development (Ohde et al., 2009; Rohith and Shyamala, 2017). In this study, the complete ORF sequence encoding Scalloped from the cigarette beetle was identified and cloned. Bioinformatic analysis showed that LsSd protein has an N-terminal TEA domain and a C-terminal YBD, which is consistent with the structures of other LsSd proteins (Yin et al., 2020; Zhang et al., 2021a). Study have found only the TEA domain of Scalloped to be critical for wing development (Srivastava et al., 2002). TEAD (the mammalian homolog of Sd) YBD recruits YAP (the mammalian homolog of Yki) to target gene promoters, where it activates gene expression *via* the Hippo-signaling pathway (Tian et al., 2010). Multiple sequence alignment and phylogenetic analysis revealed that insect Sd proteins have been highly conserved throughout evolution, with LsSd and five other Coleopteran Sd proteins forming a distinct clade.

The differential expression of the *LsSd* gene in various tissues and developmental stages suggests that *LsSd* is involved in a vast array of biological processes. Tissue-specific expression analysis revealed that *LsSd* is highly expressed in the larval brain and midgut, which is consistent with the expression of *HvSd* from *H. vigintioctopunctata* (Ohde et al., 2009). The authors speculated that *Sd* directly or indirectly regulates the expression of genes encoding hormones in the brain, and also functions in larva-to-pupa transition (Ohde et al., 2009). We also found that *LsSd* transcript levels were higher in the early pupal stage. Due to the high level of *LsSd* expression in the early pupal stage, early pupae were chosen for RNAi studies. *LsSd* knockdown produced adults with curved and wrinkled wings, which is consistent with previous studies on *Henosepilachna vigintioctopunctata* (Ohde et al., 2009), *B. mori* (Yin et al., 2020), and *L. migratoria* (Zhang et al., 2021a). Coordination between cell proliferation and cell death is essential for wing growth and development. It has been reported that the Yki-Sd complex regulates the expression of genes involved in cell growth and proliferation, including the cell cycle regulator gene *Cyclin E* (*CycE*) and the cell death inhibitor gene *Diap1* (Zhang et al., 2008; Shu and Deng, 2017). Here, we found that *LsSd* knockdown decreased the expression levels of *LsCycE* and *LsDiap1*. Vg is a target gene downstream of both (Decapentaplegic) Dpp and Wingless (Wg) signaling in *D. melanogaster*, and it plays a crucial role in the formation of the adult wing and halter structures (Fan et al., 2021). During wing development, Vg and Sd form a complex and activate the expression of genes involved in wing morphogenesis (Halder et al., 1998). In this study, knockdown of *LsSd* significantly decreased *LsVg* expression. These findings suggest that *LsSd* participates in the regulation of *L. serricorne* wing development and plays crucial roles.

Recent evidence suggests that Hippo-signaling pathway is essential for maintaining the homeostasis of the immune system (Liu et al., 2016; Yang et al., 2019). Activation of the Hippo signal inhibits production of the Yki-Sd transcription factor complex in *Drosophila*, resulting in decreased expression of *Cactus*, an inhibitor of NF-κB (IκB) in the Toll pathway (Valanne et al., 2011; Liu et al., 2016). Previous studies demonstrated that Yki silencing remarkably downregulated the expression of *Cactus* (Yang et al., 2019; Huang et al., 2022). Thus, we hypothesized that *LsSd* may be associated with innate immune in *L. serricorne*. This study demonstrated that *E. coli* infections increased *LsSd* mRNA expression, whereas *S. aureus* infections downregulated *LsSd* mRNA expression. To understand how this occurs in *L. serricorne*, we engineered *LsSd*-knockdown larvae and studied the mortality of larvae following bacterial infection. *LsSd* was confirmed as a negative regulator based on the survival analysis because its knockdown decreased susceptibility to *S. aureus*. The mortality rate of *LsSd* knockdown larvae was insignificant when infected with *E. coli*. This indicates that *LsSd* is linked to the innate immune response against *S. aureus*. Similarly, Gram-positive bacterial infection rather than Gram-negative bacteria infection acutely activates the Hippo-signaling pathway in *D. melanogaster in vivo* (Liu et al., 2016). Gram-positive and Gram-negative bacteria can activate the Hippo-signaling pathway in crabs and shrimp (Yang et al., 2019; Huang et al., 2022). In insects, the Toll signaling pathway is predominantly activated by Gram-positive bacteria (Valanne et al., 2011), whereas in crustaceans, the Toll signaling pathway is predominantly activated by both bacterial groups (Sun et al., 2017). In the present study, knockdown of *LsSd* significantly downregulated the transcription of *LsCact* in response to *S. aureus* stimulation, while upregulating the expression of five AMP genes: *LsDef1*, *LsDef2*, *LsCole*, *LsLysC*, and *LsLysI*. We further demonstrated that *LsSd* is a negative regulator in the immune defense of *L. serricorne,* by promoting the transcription of *LsCact* and negatively regulating the expression of antibacterial effectors.

In conclusion, we have confirmed that *LsSd* is involved in multiple biological processes. We confirmed that *LsSd* is essential for wing development in *L. serricorne*. Additionally, we discovered that *LsSd* is required for the innate immune-mediated host defense of *L. serricorne* larvae against *S. aureus*. Therefore, *LsSd* may play an important role in the immune response of *L. serricorne*. In the future, for *L. serricorne* control, we can develop novel pest control strategies based on the combination of RNAi of *LsSd* and microbial control.

## Data Availability

The datasets presented in this study can be found in online repositories. The names of the repository/repositories and accession number(s) can be found below: GenBank, ON533736.
