# Supplementary material for: Immune function analysis of LsSd, a transcription factor of the Hippo signaling pathway, in the cigarette beetle Lasioderma serricorne
Source: Front Physiol. 2022 Oct 11;13:1042897. doi: 10.3389/fphys.2022.1042897 (PMC9593042; doi:10.3389/fphys.2022.1042897)
Supplement: Supplementary file 1 [file DataSheet1.docx]

Supplementary Material


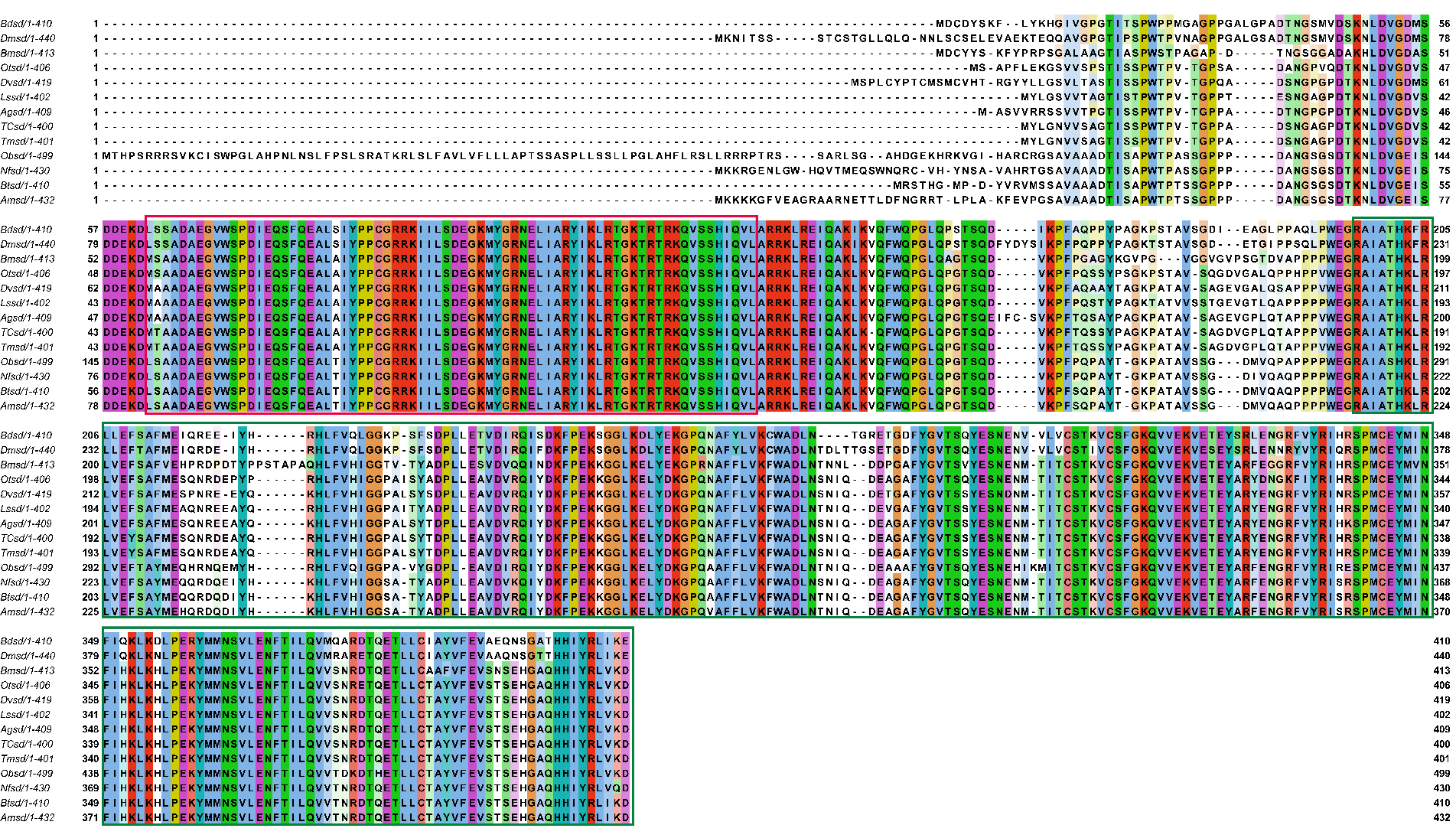


**Supplementary Figure 1.** Sequence alignment of the deduced LsSd proteins in other species homologous sequences. The DNA binding domain (TEA) and YAP-binding domain (YBD) are marked with red and green boxes respectively.

**Table S1.** Primers used in this study.

| Application of Primers | Gene Name | Forward Primer (5′-3′) | Reverse Primer (5′-3′) |
| --- | --- | --- | --- |
| ORF confirmation | *LsSd* | CATTGGAGATGTATCTAGGAAGTGT | CAGGAGCAGACTCGGATTCA |
| qRT-PCR analysis | *LsSd* | GCCCTTCCCACAATCAACATATCCT | GTGTCTCTGGTATGCTTCCTCTCTG |
|  | *LsCycE* | CCGCCGCTGTTTACTTCGTTCT | CGCCTGTTGACTCTCGGATTCG |
|  | *LsDiap1* | CCCAAGCCAAAGAAGAGCCAAGT | TCCACGCACGCAACGATATGAC |
|  | *LsVg^a^* | GGGCTGTTCACGATTACCAT | GAATCCTGAACTTGCGCTTC |
|  | *LsAtt2* | GCAGGTCCACTTACAACAGG | AGTCCTCCGCCGTAATTAGG |
|  | *LsDef1* | CGCTTTCGCTATGGTGTCTT | TGGCAGCACACAAAGAATCA |
|  | *LsDef2* | TACCCAAGCTCTTCCTGTGG | GGCAAGACAATGGAGAGCAC |
|  | *LsCole* | GAGGTCCAGACTACTCGCAG | CTGAACTCCAACCCTCCAGT |
|  | *LsLysC^b^* | CATCTCATTTTATCCGGTCATG | AGACTTAACACTTAGGTATAGTTAG |
|  | *LsLysI^b^* | CACATCCAATATGTTCGTTAAGTA | AAAAAATTCAAGACTGCAGTTG |
|  | *RPL13a* | ACCACCGTATGACCGCAGGAA | CCAGCCAACCTCGTGAGACAAC |
| dsRNA synthesis | *LsSd* | **TAATACGACTCACTATAGGG**ATCACTTATGCCGACCCTCTATT | **TAATACGACTCACTATAGGG**GCTTGCCGAACGAACAGAC |
|  | *GFP* | **TAATACGACTCACTATAGGG**CAGTTCTTGTTGAATTAGATG | **TAATACGACTCACTATAGGG**AATGTTACCATCTTCTTTAA |

a *LsVg* primer were described by Yang et al (2020).

b *LsLysC* and *LsLysI* primers were described by Yan et al (2021).

References

Yang, W. J., Xu, K. K., Yan, Y., Li, C. and Jin, D. C. (2020). Role of chitin deacetylase 1 in the molting and metamorphosis of the cigarette beetle *Lasioderma serricorne*. *Int. J. Mol. Sci.* 21, 2449.

Yan, Y., Yang, H., Xu, K. K., Hu, D. M. and Yang, W. J. (2021). Two lysozymes are involved in the larva-to-pupa transition and the antibacterial immunity of *Lasioderma serricorne* (Coleoptera: Anobiidae). *J. Stored Prod. Res.* 90, 101753.
